# N-Terminal Prosomatostatin as a Risk Marker for Cardiovascular Disease and Diabetes in a General Population

**DOI:** 10.1210/jc.2016-1736

**Published:** 2016-07-11

**Authors:** Tore Hedbäck, Peter Almgren, Peter M. Nilsson, Olle Melander

**Affiliations:** Department of Clinical Sciences, Lund University, Clinical Research Center, SE 205 02 Malmö, Sweden

## Abstract

**Context:**

Somatostatin inhibits a range of hormones, including GH, insulin, and glucagon, but little is known about its role in the development of cardiometabolic disease.

**Objective:**

The objective of the study was to investigate whether fasting plasma concentration of N-terminal prosomatostatin (NT-proSST) is associated with the development of diabetes, coronary artery disease (CAD), and mortality.

**Design, Setting, and Participants:**

NT-proSST was measured in plasma from 5389 fasting participants of the population-based study Malmö Preventive Project, with a mean baseline age of 69.4 ± 6.2 years. Cox proportional hazards models adjusted for traditional cardiovascular risk factors were used to investigate the relationships between baseline NT-proSST and end points, with a mean follow-up of 5.6 ± 1.4 years.

**Main Outcome Measures:**

CAD, diabetes, and mortality were measured.

**Results:**

Overall, NT-proSST (hazard ratio [HR] per SD increment of log transformed NT-proSST) was unrelated to the risk of incident diabetes (220 events; HR 1.05; 95% confidence interval [CI] 0.91–1.20; *P* = .531) but was related to the risk of incident CAD (370 events; HR 1.17; 95% CI 1.06–1.30; *P* = .003), all-cause mortality (756 events; HR 1.24; 95% CI 1.15–1.33; *P* < .001), and cardiovascular mortality (283 events; HR 1.33; 95% CI 1.19–1.43; *P* < .001). The relationships were not linear, with most of the excess risk observed in subjects with high values of NT-proSST. Subjects in the top vs bottom decile had a severely increased risk of incident CAD (HR 2.41; 95% CI 1.45–4.01; *P* < .001), all-cause mortality (HR 1.84; 95% CI 1.33–2.53; *P* < .001), and cardiovascular mortality (HR 2.44; 95% CI 1.39–4.27; *P* < .001).

**Conclusion:**

NT-proSST was significantly and independently associated with the development of CAD, all-cause mortality, and cardiovascular mortality.

## Abstract (precis)

In a general population, NT-proSST was measured at baseline and subjects were followed for a 5.6 years. An association between NT-proSST and incident coronary artery disease and death was found.

Although diabetes is a substantial risk factor for the development of cardiovascular disease (CVD) (1), a number of large trials of intensive glycemic control in patients with diabetes failed to reduce the risk of cardiovascular morbidity or mortality (2–4). It therefore seems plausible that there may be other mechanisms than hyperglycemia that drive the diabetes-related risk of CVD. In addition, half of patients presenting with coronary artery disease (CAD) do so with only one or none of the traditional risk factors (5). It is therefore clear that there is a need for sharper risk stratification, beyond the traditional risk factors, in the primary preventive setting.

Because of the central role of somatostatin (SST) in regulating key hormones of metabolism, it is a strong candidate for study in this regard. SST is a peptide hormone of 14 and 28 amino acid moieties, with SST-14 being predominant in the central nervous system and most peripheral organs and SST-28 mainly being produced in intestinal enteroendocrine cells (6). It is phylogenetically ancient and its precursor along with SST-14 are well preserved across vertebrate evolution (7). It acts as an inhibitor of the secretion, and thus the metabolic function, of GH, insulin, glucagon, gastrin, glucagon-like peptide, and IGF-1, among others (8, 9), several of which are implicated in either diabetes or CVD or both (10–12). Thus, we hypothesized that the somatostatin axis could be involved in the development of cardiometabolic disease and mortality.

SST's short half-life, plasma concentrations in less than the picomolar range, and the fact that it works in both an endocrine and a paracrine manner make it difficult to estimate its level of function through direct measurements in plasma (13). However, when SST is cleaved from its precursor prosomatostatin, a stable precursor fragment, N-terminal prosomatostatin [1–64] (NT-proSST), can be measured and has been shown to correspond to SST secretion (14, 15). No previous studies have investigated the relationship between circulating concentration of NT-proSST and the development of diabetes or CAD, and only one has studied its relationship with all-cause and cardiovascular mortality, which was done in a diabetic cohort rather than a general one (16).

The aim of the present study was to evaluate the association of baseline NT-proSST concentrations in plasma with the development of diabetes, CAD, all-cause mortality, and cardiovascular mortality in a prospective population-based cohort.

## Materials and Methods

### Study population

The Malmö Preventive Project is a Swedish, single-center, prospective, population-based study. Between 1974 and 1992, 33 346 individuals from the city of Malmö were recruited. The recruited subjects were screened for traditional risk factors of all-cause mortality and CVD. Between 2002 and 2006, all subjects who were alive were invited for a reexamination in which 18 240 individuals participated. The study protocols for the reexamination were approved by the Ethics Committee of Lund University (protocol number DNR 2009/633), and all participants provided written informed consent. Risk factors for diabetes and CVD were assessed, and plasma with EDTA was frozen to −80°C for later analysis. Among these 18 240 individuals, 5412 were randomly selected for the current study, and fasting NT-proSST was measured in their previously stored plasma. The only exclusion criterion was prior participation in another large, prospective study: the Malmö Diet and Cancer study. Of these 5412 individuals, 5389 had complete data on covariates used for adjustment and formed our study population. In an analysis of incident diabetes, 629 subjects with prevalent diabetes were excluded along with 222 cases discovered on the day of examination. In an analysis of incident CAD, 513 subjects with a history of CAD were excluded.

### Clinical examination and assays

Participants were interviewed and underwent a physical examination as well as a laboratory assessment. Blood pressure was measured using an oscillometric sphygmanometer twice after 10 minutes of rest in the supine position. Prevalent diabetes at the examination 2002–2006 was defined as a fasting plasma glucose level of 7.0 mmol/L or greater, a self-reported physician diagnosis of diabetes, or the use of antidiabetic medication. Cigarette smoking was elicited by a self-administered questionnaire, with current cigarette smoking defined as any use within the past year. Measurements of fasting total cholesterol, high-density lipoprotein (HDL) cholesterol, triglycerides, and glucose were made according to standard procedures at the Department of Clinical Chemistry at Malmö University Hospital. Low-density lipoprotein (LDL) cholesterol was estimated with the Friedewald equation. Serum creatinine (creatinine) was measured using the Beckman Coulter modified Jaffe procedure (17). NT-proSST was measured using an assay in the chemiluminescence/coated tube format (B.R.A.H.M.S. GmbH) (18). The assay used had a detection limit of 4 pmol/L, and the interlaboratory coefficient of variation was 20% at 18 pmol/L, 10% at 50 pmol/L, and less than 6% for above 100 pmol/L. The stability of the native analyte at 22°C and 37°C was tested in EDTA plasma from 10 different individuals. At 22°C the analyte was stable (<10% loss of immunoreactivity) for 72 hours, and at 37°C, the analyte was stable for 24 hours. Samples were analyzed in duplicate. Prolonged frozen storage and repeated freeze-thaw cycles had no bearing on the measured concentration of NT-proSST (mean values 99.1% [range 93.8%–104.3%] of the original values).

### Follow-up and end point retrieval

Subjects were followed up for incident diabetes, incident CAD events, all-cause mortality, and cardiovascular mortality until December 31, 2010. The mean follow-up time was 5.6 years. CAD events and deaths, including cause of death, were identified by linking a 10-digit Swedish personal identification number with three registers: the Swedish Hospital Discharge Register, the Swedish Cause of Death Register, and the Swedish Coronary Angiography and Angioplasty Registry. These registers have been previously described and validated for classifications of outcomes (19, 20). CAD was defined as coronary artery revascularization, any myocardial infarction, or death due to ischemic heart disease. Myocardial infarction was defined on the basis of *International Classification of Diseases*, 9th revision (ICD-9), code 410 or *International Classification of Diseases*, 10th revision (ICD-10) code I21. Death attributable to ischemic heart disease was defined as ICD-9 codes 412 and 414 or ICD-10 codes I22, I23, or I25. Coronary artery bypass surgery was identified from national Swedish classification systems of surgical procedures and defined as procedure codes 3065, 3066, 3068, 3080, 3092, 3105, 3127, or 3158 in the Op6 system or as procedure code FN in the KKÅ97 system. Percutaneous intervention was identified from the Swedish Coronary Angiography and Angioplasty Registry. Cardiovascular mortality was defined as primary cause of death classified as ICD-9 codes 390–459 and ICD-10 codes I00-I99.

New-onset diabetes cases were retrieved from six different national and regional diabetes registers: individuals could be registered as having a diagnosis of diabetes in the nationwide Swedish National Diabetes Register (21) or in the regional Diabetes 2000 register of the Scania region of which Malmö is the largest city (22) or in the Swedish National Patient register, which is a principal source of data for numerous research projects that covers more than 99% of all somatic and psychiatric hospital discharges and Swedish hospital-based outpatient care (19), or they could be classified as diabetes cases if they had diabetes as a cause of death in the Swedish Cause of Death Register, which comprises all deaths among Swedish residents occurring in Sweden or abroad (23) or if they had been prescribed antidiabetic medication as registered in the Swedish Prescribed Drug Register (24) or if they had at least two glycated hemoglobin (HbA1c) recordings of 6.0% or greater using the Swedish Mono-S standardization system, corresponding to 7.0% or greater according to the US National Glycohemoglobin Standardization Program (NGSP), in the Malmö HbA1c register, which has analyzed and catalogued all HbA1c samples at the Department of Clinical Chemistry taken in institutional and noninstitutional care in the greater Malmö area from 1988 onward.

### Statistics

The distribution of plasma NT-proSST concentrations was skewed and therefore logarithmically transformed. NT-proSST was then related to risk of development of diabetes, CAD, all-cause mortality, and cardiovascular mortality using multivariate adjusted Cox proportional hazards models and Kaplan-Meier plots. All Cox proportional hazards models were adjusted for age, gender, body mass index (BMI), HDL cholesterol, LDL cholesterol, systolic blood pressure (BP), antihypertensive therapy, current smoking, and diabetes except the model for analysis of incident diabetes, which was adjusted for a fasting plasma glucose level instead of prevalent diabetes. The CAD and mortality analyses were also run a second time using creatinine as a covariate in addition to the established cardiovascular risk factors stated above. Logarithmically transformed creatinine was used due to a skewed distribution. The proportional hazards assumption was examined using Schoenfeld residuals. Multiple linear regression was performed, with NT-proSST being the dependent variable and with age, gender, BMI, HDL cholesterol, LDL cholesterol, systolic BP, antihypertensive therapy, current smoking, and diabetes being the explanatory variables. The additional value of NT-proSST in risk prediction was assessed using both Harrell's C statistics and the continuous net reclassification improvement. Statistical analysis was performed using SPSS (version 22.0; IBM Corp) and STATA (release 13; StataCorp). A two-sided value of *P* < .05 was considered significant.

## Results

At baseline, the study population comprised 5389 individuals, the mean age was 69 years, and 70% were males (Table 1). The mean follow-up time was 5.6 ± 1.4 years. In the study population free from prevalent diabetes (n = 4760), 220 incident cases of diabetes were recorded. In the study population without a history of CAD (n = 4876), 370 incident CAD events were recorded. In the entire study population, 756 deaths occurred, 283 of which were attributable to cardiovascular causes.

**Table 1.** Study Baseline Characteristics

Characteristic	Baseline Value (n = 5389)
Age, y	69.4 ± 6.2
Gender, n, % men	3758 (69.7%)
BMI, kg/m^2^	27.2 ± 4.2
HDL cholesterol, mmol/L	1.4 ± 0.4
LDL cholesterol, mmol/L	3.6 ± 1.0
Systolic BP, mm Hg	146 ± 21
Diastolic BP, mm Hg	84 ± 11
Antihypertensive therapy, n, %	2128 (39.5%)
Current smoking, n, %	1056 (19.6%)
Prevalent diabetes mellitus, n, %^b^	851 (15.8%)
History of CAD, n, %	513 (9.5%)
NT-proSST, pmol/L^a^	439 (351–565)

All values are expressed as mean ± SD except as otherwise noted.

a NT-proSST concentration is expressed as median (interquartile range).

b Cases discovered of the day of examination included.

Multiple linear regression showed significant and independent relationships between NT-proSST and all explanatory variables except for HDL cholesterol, LDL cholesterol, and systolic BP (Table 2). These variables were still included as covariates in the Cox regression analyses because they are widely considered traditional risk factors in the metabolic and cardiovascular disease panoramas. The significant relationships all had positive β-coefficients in relation to NT-proSST, except for BMI.

**Table 2.** Independent Determinants of Fasting Plasma NT-proSST Concentration From a Multiple Linear Regression Model

Independent Determinant of NT-proSST	β-Coefficient (95% CI)^a^	*P* Value
Age, per year	0.03 (0.02–0.03)	<.001
Gender (female)	0.08 (0.02–0.14)	.01
BMI, per kg/m^2^	−0.02 (−0.03 to −0.02)	<.001
HDL cholesterol, per mmol/L	−0.03 (−0.10 to 0.05)	.46
LDL cholesterol, per mmol/L	−0.01 (−0.04 to 0.02)	.51
Systolic BP, per 10 mm Hg	−0.01 (−0.02 to 0.00)	.09
Antihypertensive therapy	0.24 (0.18–0.29)	<.001
Current smoking	0.50 (0.44–0.57)	<.001
Prevalent diabetes mellitus	0.24 (0.16–0.32)	<.001

a β-Coefficient is expressed as the increment of the logarithmically transformed standardized values of NT-proSST per increment of standardized values (or presence of dichotomized risk factor) of the risk factor in question.

**Figure 1.**
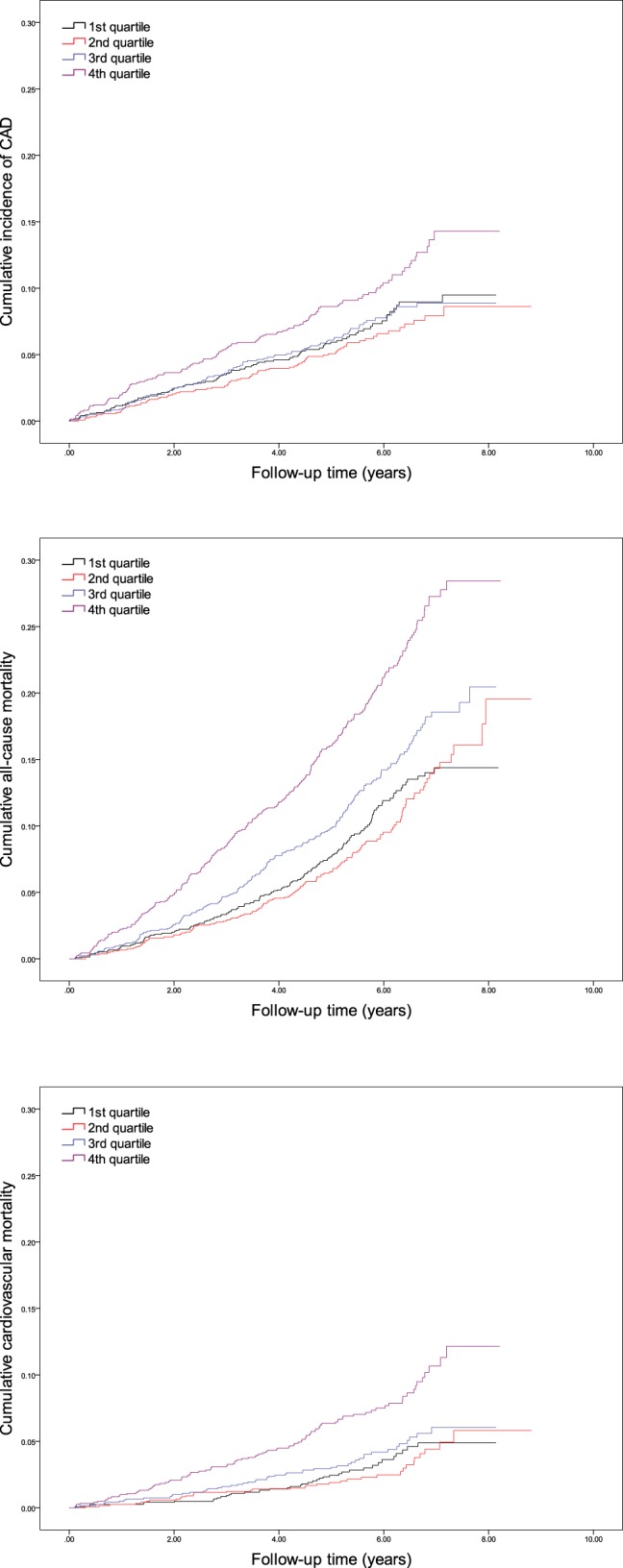
Kaplan-Meier failure estimates for primary end points of CAD, all-cause mortality, and cardiovascular mortality, according to quartiles of baseline fasting plasma concentration of NT-proSST.

The associations between end points and concentrations of NT-proSST were analyzed looking at continuous per SD increments of logarithmically transformed NT-proSST as well as per quartile (Figure 1 and Table 3). There was no significant relationship between NT-proSST and incident diabetes. For CAD, all-cause mortality, and cardiovascular mortality, there were significant and independent associations in continuous analysis of NT-proSST, with hazard ratios of 1.17 (95% confidence interval [CI] 1.06–1.30), 1.24 (95% CI 1.15–1.33), and 1.33 (95% CI 1.19–1.43) per SD increment of NT-proSST, respectively. When considering the fourth quartile of NT-proSST concentration, there was no significant association in relation to the incidence of CAD. However, subjects in the fourth quartile had hazard ratios of 1.39 (95% CI 1.13–1.72) and 1.75 (95% CI 1.23–2.48), relative to subjects in the first quartile, to die and to die from cardiovascular causes, respectively, but there was no significant excess risk in subjects belonging to the second or third quartiles in comparison with subjects belonging to first quartile. When examining the hazard ratios in the quartile analysis, it appears as if the relationships between NT-proSST and CAD, all-cause mortality, and cardiovascular mortality are not linear (Table 3). This nonlinearity becomes even more evident when considering deciles of NT-proSST concentration: in the 10th decile, the hazard ratios relative to the first decile are 2.41 (95% CI 1.45–4.01) for CAD, 1.84 (95% CI 1.33–2.53) for all-cause mortality, and 2.44 (95% CI 1.39–4.27) for cardiovascular mortality (Table 4). There was no consistent linear trend at more central parts of the NT-proSST distribution.

**Table 3.** Fasting Plasma Concentration of NT-proSST in Relation to Future Risk of Diabetes, CAD, All-Cause Mortality, and Cardiovascular Mortality in the Malmö Preventive Project

	All Patients	*P* Value	Quartile 1	Quartile 2	Quartile 3	Quartile 4	*P* for Trend
Diabetes							
n/n cases^a^	4538/220		1130/59	1132/45	1138/52	1138/64	
NT-proSST, pmol/L^b^	436 (72–2680)		303 (72–349)	392 (350–435)	484 (436–555)	666 (556–2680)	
HR (95% CI)^c^	1.05 (0.91–1.20)	0.531	1.0 (referent)	0.79 (0.54–1.17)	0.94 (0.64–1.37)	1.08 (0.75–1.55)	.671
CAD							
n/n cases^a^	4876/370		1218/85	1226/76	1211/87	1221/122	
NT-proSST, pmol/L^b^	436 (72–3620)		301 (72–348)	393 (349–436)	486 (437–556)	666 (557–3620)	
HR (95% CI)^c^	1.17 (1.06–1.30)	.003	1.0 (referent)	0.89 (0.66–1.22)	0.97 (0.71–1.31)	1.24 (0.92–1.65)	.108
All-cause mortality							
n/n cases^a^	5389/756		1349/142	1340/134	1352/191	1348/289	
NT-proSST, pmol/L^b^	439 (72–3620)		302 (72–351)	395 (352–438)	490 (439–564)	680 (565–3620)	
HR (95% CI)^c^	1.24 (1.15–1.33)	<.001	1.0 (referent)	0.84 (0.66–1.07)	1.08 (0.87–1.34)	1.39 (1.13–1.72)	<.001
Cardiovascular mortality							
n/n cases^a^	5389/283		1349/47	1340/49	1352/65	1348/122	
NT-proSST, pmol/L^b^	439 (72–3620)		302 (72–351)	395 (352–438)	490 (439–564)	680 (565–3620)	
HR (95% CI)^c^	1.33 (1.19–1.43)	<.001	1.0 (referent)	0.94 (0.63–1.41)	1.10 (0.75–1.61)	1.75 (1.23–2.48)	<.001

Abbreviations: HR, hazard ratio; CAD, coronary artery disease.

a n/n cases refer to number of participants per number of incident cases of diabetes, CAD, all-cause deaths, and cardiovascular deaths.

b NT-proSST concentration is expressed as median (range).

c HRs (95% CI) are expressed per SD increment of logarithmically transformed NT-proSST. In analyses of quartiles, the lowest quartile (quartile 1) was defined as the reference category and the HR (95% CI) for each of quartiles 2, 3, and 4 were compared with the reference quartile. Analyses were adjusted for age, gender, BMI, HDL cholesterol, LDL cholesterol, systolic BP, antihypertensive therapy, current smoking, and diabetes except the diabetes model, which was adjusted for plasma glucose levels instead of diabetes.

**Table 4.** Fasting Plasma Concentration of NT-proSST in Deciles, in Relation to Future Risk of CAD, All-Cause Mortality, and Cardiovascular Mortality in the Malmö Preventive Project

Decile of NT-proSST	CAD, n/n Cases^a^	CAD HR (95% CI)^b^	All-Cause Mortality, n/n cases^a^	All-Cause Mortality HR (95% CI)^b^	Cardiovascular Mortality, n/n cases^a^	Cardiovascular Mortality HR (95% CI)^b^
First	481/21	1.0 (referent)	539/53	1.0 (referent)	539/16	1.0 (referent)
Second	489/42	2.01 (1.23–3.52)	537/66	1.32 (0.92–1.89)	537/24	1.64 (0.87–3.10)
Third	493/40	2.02 (1.19–3.43)	538/43	0.78 (0.52–1.17)	538/17	1.04 (0.53–2.07)
Fourth	483/31	1.55 (0.89–2.70)	548/46	0.81 (0.54–1.20)	548/15	0.90 (0.45–1.83)
Fifth	498/27	1.29 (0.73–2.28)	527/68	1.18 (0.82–1.69)	527/24	1.43 (0.76–2.69)
Sixth	488/43	2.01 (1.19–3.40)	543/77	1.26 (0.88–1.78)	543/27	1.48 (0.80–2.76)
Seventh	477/25	1.15 (0.64–2.07)	538/77	1.22 (0.86–1.73)	538/22	1.16 (0.61–2.22)
Eighth	490/43	1.95 (1.15–3.30)	543/73	1.07 (0.75–1.53)	543/33	1.62 (0.89–2.96)
Ninth	489/38	1.63 (0.95–2.79)	537/103	1.43 (1.03–2.01)	537/44	2.03 (1.14–3.63)
10th	488/60	2.41 (1.45–4.01)	539/150	1.84 (1.33–2.53)	539/61	2.44 (1.39–4.27)

Abbreviation: CAD, coronary artery disease; HR, hazard ratio.

a n/n cases refer to number of participants per number of incident cases of CAD, all-cause deaths, and cardiovascular deaths.

b The lowest decile (decile 1) was defined as the reference category, and the HR (95% CI) for each decile was compared with the reference decile. Analyses were adjusted for age, gender, BMI, HDL cholesterol, LDL cholesterol, systolic BP, antihypertensive therapy, current smoking, and diabetes.

The Cox regression analyses for CAD and mortality end points were also done using a model adjusting for creatinine in addition to the covariates in the basic model. Some individuals were excluded from this model due to missing values of creatinine, 209 of 4876 missing for the CAD end point, and 234 of 5389 missing for mortality end points. In a continuous analysis of the model including creatinine, hazard ratios per SD increment were 1.17 (95% CI 1.04–1.31) for CAD, 1.23 (95% CI 1.13–1.33) for all-cause mortality, and 1.35 (95% CI 1.19–1.53) for cardiovascular mortality, virtually the same results as for the model without creatinine.

To assess NT-proSST's usefulness in risk prediction, Harrell's C statistics, which is an iteration on receiver-operating characteristic analysis adapted for survival analysis and approximately should be interpreted as the area under the curve in receiver-operating characteristic space, was used (25). A comparison was made between a model using the same traditional risk factors as in the Cox regression analyses and a model that additionally included NT-proSST in addition to traditional risk factors. For CAD, the C statistic was 0.67 95% CI (0.64–0.70) for the basic model and 0.68 (95% CI 0.65–0.70) for the model including NT-proSST; for all-cause mortality, the C statistic was 0.72 (95% CI 0.70–0.74) for the basic model and 0.73 (95% CI 0.71–0.75) for the model including NT-proSST; and finally for cardiovascular mortality, the C statistic was 0.75 (95% CI 0.73–0.78) for the basic model and 0.76 (95% CI 0.74–0.79) for the model including NT-proSST, all of which show only marginal differences in discrimination between the models with and without NT-proSST. When adding NT-proSST in addition to the traditional risk factors, however, the continuous net reclassification improvement was significant for both all-cause mortality (0.15, *P* = .01) and cardiovascular mortality (0.20, *P* = .01) but not for CAD (0.05, *P* = .49).

## Discussion

The key findings of this study are that fasting plasma concentration of NT-proSST independently predicts development of CAD and both all-cause and cardiovascular mortality but not diabetes. The significant associations are not linear: the upper quartiles and deciles seem to drive the overall association with the three end points. The effect size of the association is greatest between NT-proSST and cardiovascular mortality, followed by all-cause mortality, followed by CAD.

One of the aims of this study was to look for a link between SST and diabetes, and although the presence of diabetes at the baseline examination was related in a cross-sectional manner, significantly and independently, to higher concentrations of NT-proSST, the Cox regression analysis showed no significant association between NT-proSST levels and later development of new-onset diabetes. Given how SST plays a major role in the homeostasis of glucose metabolism, inhibiting the release of insulin, glucagon, and incretins, along with the fact that hyperglycemia is a known side effect of the administration of somatostatin analogues (26), this was somewhat surprising. However, the interindividual differences in circulating concentration of somatostatin, reflected by variation in the fasting concentration of NT-proSST in the study population, are quite small compared with the unphysiological amounts that are required to be administered for iatrogenic hyperglycemia to arise. Although NT-proSST did correlate in a cross-sectional manner with diabetes prevalence, the lack of association with diabetes incidence suggests that this may represent a secondary or temporary phenomenon rather than a primary process promoting diabetes development.

As mentioned, there is one previous investigation of the relationship between NT-proSST and mortality end points, in persons with diabetes participating in the Zwolle Outpatient Diabetes project Integrating Available Care prospective cohort study (16). It found that when including creatinine levels as a covariate in the Cox regression analysis, the independent relationship between NT-proSST and mortality end points vanished. In our study, adjusting for creatinine levels does not eliminate independence of NT-proSST as a variable. Speculatively, it is possible that the connection between creatinine and NT-proSST is exaggerated in a population of persons with diabetes due to the high prevalence of renal failure in such a population. The ZODIAC study also found no change in the C statistic when adding NT-proSST on top of traditional risk factors, whereas there was a marginal C statistic change in this study. Regardless of this fact, it has been previously shown that it is problematic to put too much emphasis on the C statistic alone in the evaluation of prediction models and that looking at the continuous net reclassification improvement can be more sensitive in model comparisons in which the traditional model already is relatively fine-tuned, which is the case here (27, 28). The significant net reclassification improvement found in this study indicates that using a model that incorporates NT-proSST, in addition to traditional risk factors, can significantly reclassify cases when it comes to predicting mortality.

The association between NT-proSST and incident CAD deserves special mention. In continuous analysis we observed a statistically significant relationship, whereas there was no linear trend either across quartiles or deciles of NT-proSST. Although decile analyses are difficult to draw firm conclusions from due to the small number of events within each decile, one explanation for the significant association between continuous NT-proSST and incident CAD is that it is in fact nonlinear and mainly driven by subjects with the highest levels, such as those in the top decile, who had significantly higher risk of CAD when compared with the lowest decile of NT-proSST. In fact, despite significant linear trends over NT-proSST quartiles in relation to all-cause mortality and cardiovascular mortality, only subjects belonging to the fourth quartile had a significantly higher risk when compared with those in the first quartile. Taken together, whether elevation of NT-proSST reflects a causative role of the somatostatin system in cardiovascular morbidity and mortality or whether it is just a consequence of another pathophysiological process, there seems to be a threshold of NT-proSST at which the risk starts to increase rather than there being a continuum of risk. In addition, not only does the NT-proSST relationship with incident CAD appear to be weaker than that with mortality, but also the concentration threshold at which the risk starts to increase seems to start at higher NT-proSST concentrations for incident CAD than for both all-cause mortality and cardiovascular mortality.

There are several weaknesses of this study to acknowledge. Crucially, as with all observational epidemiological studies, the analyses performed can show only association, and not causality, between NT-proSST and end points. Speculatively, several different scenarios are possible: SST may be directly and causally related to CAD and cardiovascular mortality, or it may mediate the risk through one or more of the risk factors with which it was correlated (such as smoking), or it may simply correlate with one or more distinct pathophysiological process. As a consequence, even if the association between NT-proSST and cardiovascular morbidity and mortality was independent of all traditional cardiovascular risk factors, as well as renal function, no conclusions regarding the pathophysiological role of SST can be drawn from the results of this study. Additionally, our study population is likely to be healthier, and thus not fully representative of a general population, compared with the average population of the corresponding age because all subjects survived from the initial examination of the Malmö Preventive Project in 1974–1992, which can be considered a selection bias. Also, with the age of the study population being high, a relatively large amount of subjects had to be excluded from statistical analysis due to the diseases already being prevalent in the population. Lastly, the assay used only targets N-terminal fragments of proSST that are present in peripheral venous blood, and thus, the paracrine effects of SST that depend on where in the body it is being secreted cannot be taken into account. However, high levels of NT-proSST detected by this assay is likely related to an overall increase in SST tone. In our opinion, the most promising candidate mechanism for such an increase in tone would be an increase in postprandial gastrointestinal secretion of SST-28 related to diet. This is in light of several studies previously suggesting that the postprandial secretion of SST-28 depends on the fat content of the meal and that SST-28 secretion is known to inhibit both the exocrine and the endocrine pancreas (29, 30), whereas the SST-14 tone seems to remain fairly constant outside the case of acromegaly (31).

In our opinion there are at least two potential fields of research that warrant further investigation. First, if SST turns out to be a causal factor in the pathology driving the increase in mortality observed in this study, there is a possibility of inhibiting its effects pharmacologically, so mechanistic studies as well as Mendelian randomization studies are warranted to assess causality. Second, and regardless of causality, this study has shown that incorporating NT-proSST into traditional models significantly reclassifies patients for cardiovascular mortality outcomes. This could be of future clinical use, allowing clinicians to more accurately select which patients would benefit from primary prevention in the form of statins and which would not.

In conclusion, NT-proSST significantly and independently predicts CAD, all-cause mortality, and cardiovascular mortality in a general population. Our results warrant more research on its role in risk stratification as well as on a possible pathophysiological involvement of the SST system in cardiovascular morbidity and mortality.
